# Supplementary material for: Maternal High-Fat Diet Aggravates Allergic Asthma in Offspring via Modulating CD4+ T-Cell Differentiation
Source: Nutrients. 2022 Jun 16;14(12):2508. doi: 10.3390/nu14122508 (PMC9230034; doi:10.3390/nu14122508)
Supplement: Supplementary file 1 [file nutrients-14-02508-s001.zip › Supplementary tables.pdf]

Table S1. Component of high-fat diet

Component	Gm%	Kcal%
Protein	26.2	20
Carbohydrate	26.3	20
Fat	34.9	60
Total（kcal/gm）	5.24	100
Component of high fat diet:		
Component	Gm	Kcal
Casein, 80 Mesh	200	800
L-Cystine	3	12
Corn Starch	0	0
Maltodextrin 10	125	500
Sucrose	68.8	275.2
Cellulose, BW200	50	0
Soybean Oil	25	225
Lard	245	2205
Mineral Mix, S10026	10	0
DiCalcium Phosphate	13	0
Calcium Carbonate	5.5	0
Potassium Citrate, 1 H2O	16.5	0
Vitamin Mix, V10001	10	40
Choline Bitartrate	2	0
FD&C Blue Dye	0.05	0
Total	773.85	4057

Typical analysis of cholesterol in lard = 0.95 mg/gram.

Table S2. **Sequences for real-time PCR primers in this study**

Gene name	Sequences (5'-3')
Mouse *Rpl13a*-F	GGGCAGGTTCTGGTATTGGAT
Mouse *Rpl13a*-R	GGCTCGGAAATGGTAGGGG
Mouse *Il-4*-F	TTGTCAT CCTGCTCTTCTTTCTC
Mouse *Il-4*-R	CAGGAAGTCTTTCAGTGATGTGG
Mouse *Il-5*-F	CTCTGTTGACAAGCAATGAGACG
Mouse *Il-5*-R	TCTTCAGTATGTCTAGCCCCTG
Mouse *Il-10*-F	GCCAAGCCTTATCGGAAATG
Mouse *Il-10*-R	TCTTCACCTGCTCCACTG
Mouse *Il-13*-F	CCTGGCTCTTGCTTGCCTT
Mouse *Il-13*-R	GGTCTTGTGTGATGTTGCTCA
Mouse *Ifng*-F	ATGAACGCTACACACTGCATC
Mouse *Ifng*-R	CCATCCTTTTGCCAGTTCCTC
Mouse *klf2*-F	GAGCCTATCTTGCCGTCCTTT
Mouse *klf2*-R	CACGTTGTTTAGGTCCTCATCC
Mouse *p53*-F	CTCTCCCCCGCAAAAGAAAAA
Mouse *p53*-R	CGGAACATCTCGAAGCGTTTA
Mouse *hif1α*-F	ACCTTCATCGGAAACTCCAAAG
Mouse *hif1α*-R	CTGTTAGGCTGGGAAAAGTTAGG
Mouse *cyclind2*-F	GAGTGGGAACTGGTAGTGTTG
Mouse *cyclind2*-R	CGCACAGAGCGATGAAGGT
Mouse *cyclind3*-F	CGAGCCTCCTACTTCCAGTG
Mouse *cyclind3*-R	GGACAGGTAGCGATCCAGGT
Human *β-actin-F*	CCAACCGCGAGAAGATGA
Human *β-actin-R*	CCAGAGGCGTACAGGGATAG
Human *Il-4*-F	CCAACTGCTTCCCCCTCTG
Human *Il-4*-R	TCTGTTACGGTCAACTCGGTG
human *Il-5*-F	TGGAGCTGCCTACGTGTATG
human *Il-5*-R	TTCGATGAGTAGAAAGCAGTGC
human *Il-13*-F	GAGGATGCTGAGCGGATTCTG
human *Il-13*-R	CACCTCGATTTTGGTGTCTCG

human *Ifng*-F	TCGGTAACTGACTTGAATGTCCA
human *Ifng*-R	TCGCTTCCCTGTTTTAGCTGC

Table S3. **Sequences for mouse bisulfite PCR primers in this study**

Gene name	Sequences (5'-3')
Il4 promoter 1 -F	TTGTAAGATTAGTTGGTTTAGGATG
Il4 promoter 1 -R	TTTCAACATAAAAAATTACACCATA
Il4 promoter 2 -F	GTTAGTATTGTATTGTTAGTATTTTTTGAT
Il4 promoter 2 -R	ATCTCTTAAACTTTATCCCTAATCCTA
Il4 CNS 1 -F	TATAAGGGTTGAGGAAGAGTAATGTAGTTTTTATAT
Il4 CNS 1 -R	CACTTTAAACTAATCAAACCTAAAACTATATCCATA
Il4 intron 1 and exon 2-F	TGGTTGAGGTAGGATTAGGGATAAAG
Il4 intron 1 and exon 2-R	CCTCCAAATATACCACAACAAACC

Table S4. **Sequences for human bisulfite PCR primers in this study**

Gene name	Sequences (5'-3')
Il4 promoter 1 -F	AAATTTATAGGGAGGTTTAGGTATAGTG
Il4 promoter 1 -R	TTCTCCTACCTCAACCTCCCCRAATAACTA
Il4 promoter 2 -F	GGTTTGATTTTATAGGAATATTTTATTTGTTTGTG
Il4 promoter 2 -R	CCCATTAATAAATATCRATTTACAATAACAATATAAAAC
Il4 promoter 3 -F	TTTAGTAAATGGGGAGATTTATTTTTAAATGTTTG
Il4 promoter 3 -R	AACCAAAACATACTTAAAAATAAAAATCAAACTTC

Table S5. Comparison of maternal and neonatal variables

	CON n(%)	HC n(%)	P value
Maternal			
Age	31.05±3.02	30.83±3.45	0.84
Education background			0.56
High school	1(5.00)	3(16.67)	
Undergraduate	16(80.00)	13(72.22)	
Graduate	3(15.00)	2(11.11)	
Height (cm)	162.90±5.18	161.11±3.63	0.23
Pre-pregnancy weight (kg)	55.65±7.15	57.22±4.99	0.44
Pre-pregnancy BMI	20.91±1.80	22.02±1.60	0.05
Gestational weight gain (kg)	13.97±4.40	16.25±4.04	0.11
Prenatal BMI	26.14±2.93	28.27±2.56	0.02*
Gravidity			0.92
1	8(40)	7(38.89)	
2	6(30)	4(22.22)	
≥3	6(30)	7(38.89)	
Parity			0.34
1	10(50.00)	12(66.67)	
2	10(50.00)	6(33.33)	
Neonatal			
Gestational age	38.98±0.75	39.35±1.01	0.18
Birthweight (kg)	3.57±0.48	3.71±0.51	0.37
Birth length (cm)	50.35±1.50	50.78±1.40	0.37
Sex			0.52
Male	11(55.00)	12(66.67)	
Female	9(45.00)	6(33.33)	

Note: Data were summarized as mean ± SEM. Categorical variables are expressed as a frequency with a proportion. *P* values were determined by two-tailed Student's *t* test or Fisher’s exact test, *$P<0.05$.
