# Supplementary material for: Maternal High-Fat Diet Aggravates Allergic Asthma in Offspring via Modulating CD4+ T-Cell Differentiation
Source: Nutrients. 2022 Jun 16;14(12):2508. doi: 10.3390/nu14122508 (PMC9230034; doi:10.3390/nu14122508)
Supplement: Supplementary file 1 [file nutrients-14-02508-s001.zip › Supplementary figures.pdf]

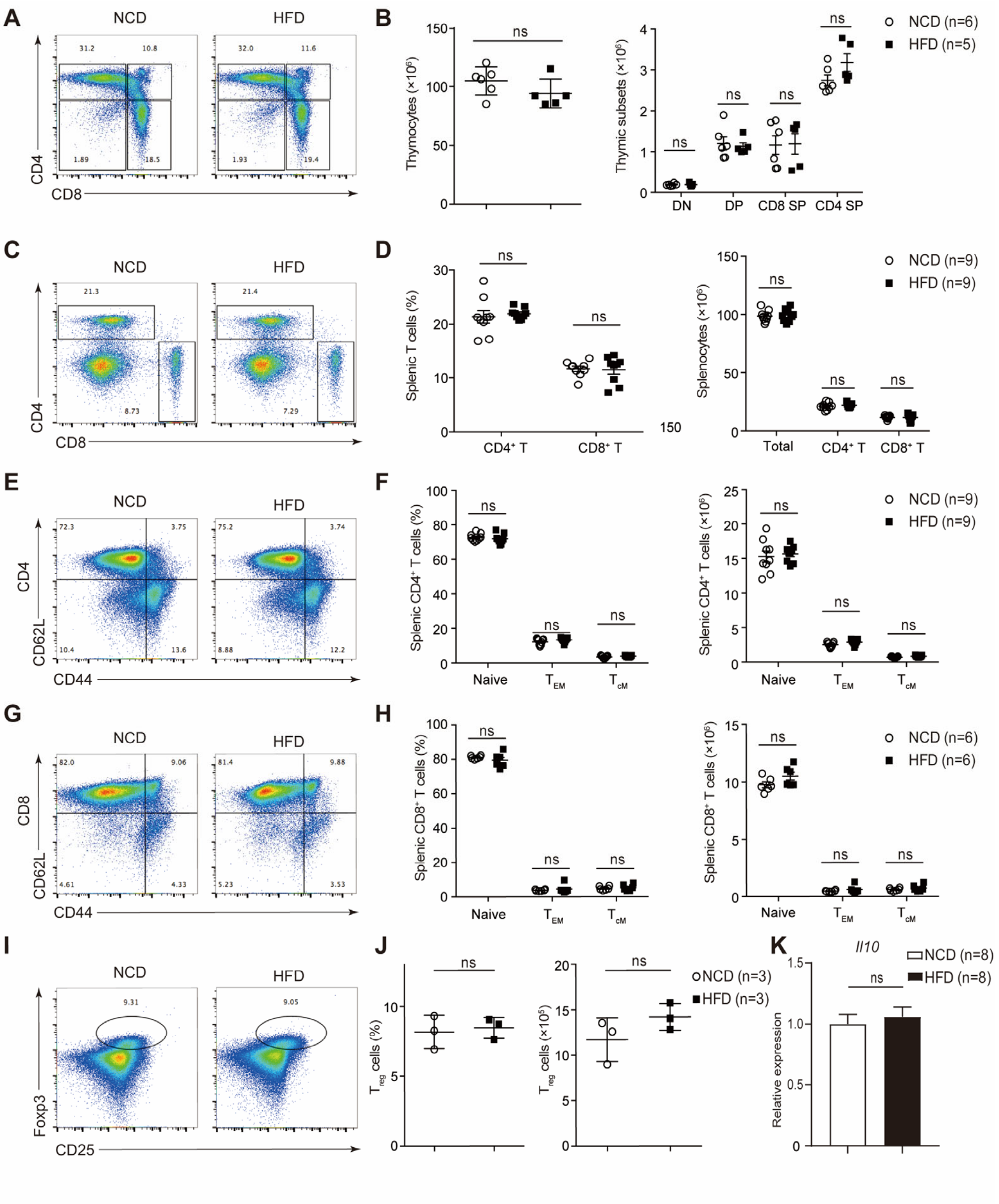

Figure S1. Maternal high-fat diet during pregnancy did not disturb T-cell homeostasis in thymus and periphery in offspring under the steady state. Flow cytometry (**A**), proportion and number (**B**) of thymocytes in NCD and HFD mice. Flow cytometry (**C**), proportions and numbers (**D**) of CD4$^+$ and CD8$^+$ T-cells in the spleens. Proportions and numbers of naïve (CD44^{lo}CD62L^{hi}), effector memory (T$_{EM}$, CD44^{hi}CD62L^{lo}) and central memory (T$_{CM}$, CD44^{hi} CD62L^{hi}) cells in CD4$^+$ (**E**,**F**) and CD8$^+$ (**G**,**H**) T-cells in spleens. Flow cytometry (**I**), proportions and numbers (**J**) of CD25$^+$ Foxp3$^+$ T$_{reg}$ cells gated from splenic CD4$^+$ T-cells. (**K**) *Il-10* mRNA expression level of splenic naïve CD4$^+$ T-cells. *p* values were determined by a Student's *t*-test. Each dot indicates a value acquired from a single mouse. Data are presented as means ± SEM. *n*, Number of mice in each group. *ns*, not significant.

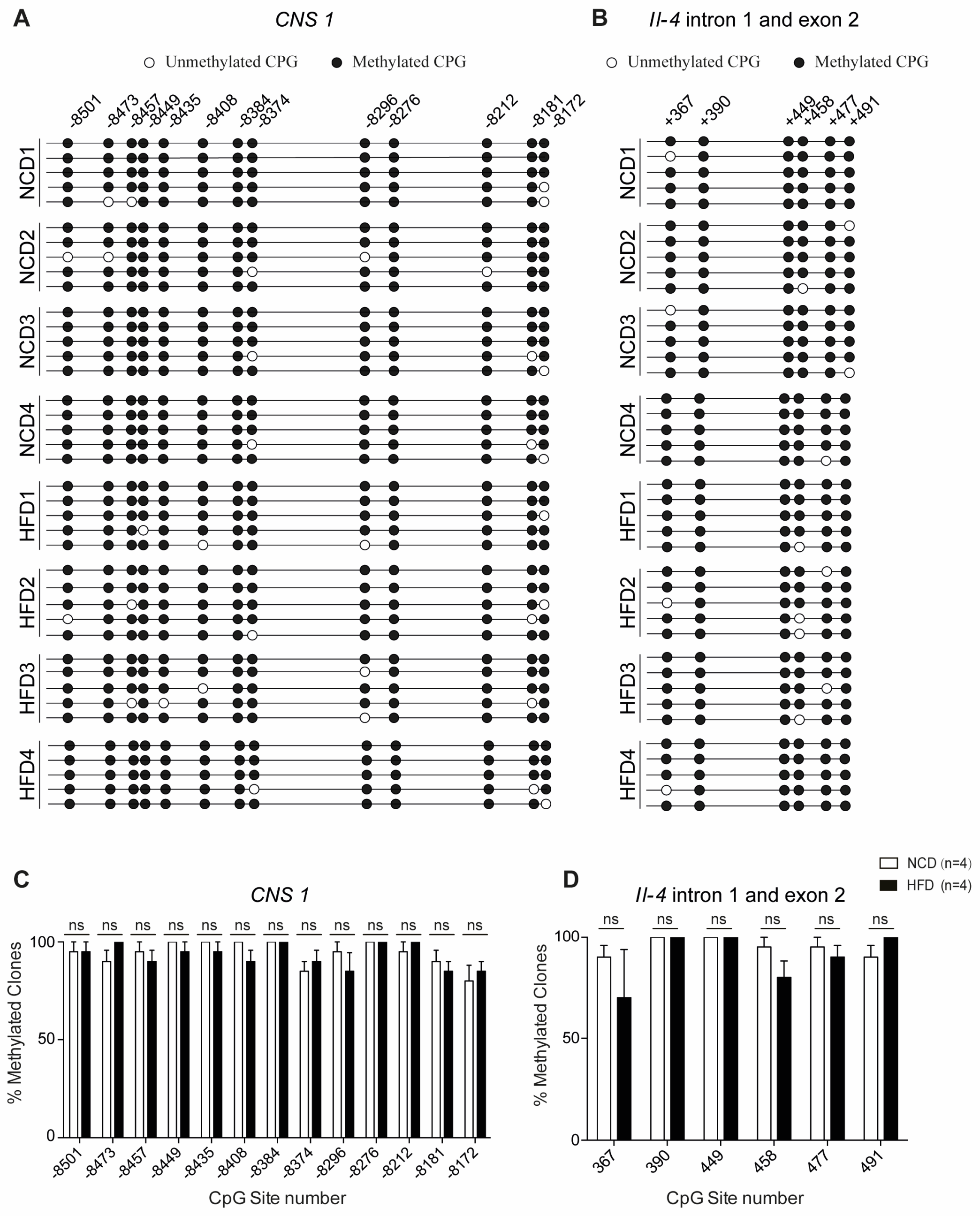

Figure S2. Regulatory regions of TH2 cytokine locus *CNS 1*, *Il-4* intron 1 and exon 2 were not differentially methylated in NCD and HFD naïve CD4+ T-cells. Naïve CD4+ T-cells were assessed for *Il-4* promoter region methylation via bisulfite sequencing (**A**-**D**). Representative data and quantifications of methylation at CpG sites of the *CNS 1* (**A**,**C**) and *Il-4* intron 1 and exon 2 locus (**B**,**D**) in naïve CD4+ T cells were shown. *p* values were determined by Student's *t*-test. Data are shown as means ± SEM. *n*, Number of mice in each group. *ns*, not significant.

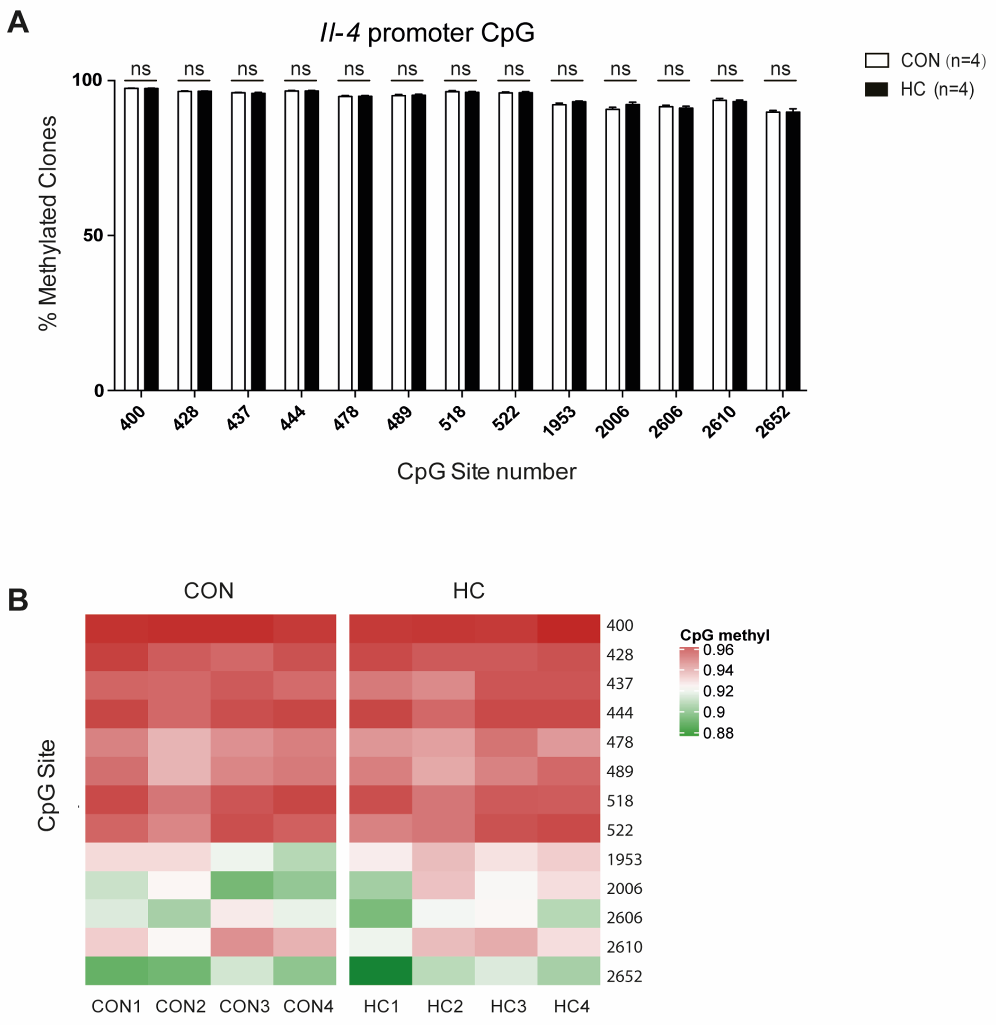

Figure S3. Methylation level of *Il-4* promoter region were comparable in cord blood CD4+ T-cells between CON and HC. CD4+ T-cells were isolated from cord blood and assessed for methylation status in *Il-4* promoter locus via targeted bisulfite sequencing. (**A**,**B**) Quantifications of methylation at CpG sites of the *Il-4* promoter region. *ns*, not significant.
